# Is the feeling of ‘weakness’ associated with unhelpful thoughts or distress regarding symptoms?

**DOI:** 10.1177/17531934241274134

**Published:** 2024-08-22

**Authors:** Floor A. Davids, Niels Brinkman, Melle M. Broekman, David Ring, Lee M. Reichel, Gregg A. Vagner

**Affiliations:** Department of Surgery and Perioperative Care, Dell Medical School, The University of Texas at Austin, 1601 Trinity St, Austin, TX 78712, United States of America.

**Keywords:** Upper extremity, non-trauma, weakness, incapability

## Abstract

This cross-sectional study looked for factors associated with feelings of weakness, level of capability and pain intensity in people seeking musculoskeletal speciality care for non-traumatic upper extremity conditions. A survey was conducted in 139 English-speaking adults, with 135 participants completing it. We found that greater intensity of feelings of weakness correlated with higher distress regarding symptoms and with older age. Lower level of capability was associated with greater intensity of feelings of weakness, greater distress regarding symptoms and older age. Higher pain intensity was associated with greater distress regarding symptoms and greater intensity of feelings of weakness. These findings suggest that the symptom of weakness may be a cue to explore potential distress about symptoms in addition to examining for actual weakness. This understanding could be a guide to a more compassionate approach to alleviate distress rather than focusing on neuromuscular pathophysiology alone, with the potential to reduce unnecessary tests and treatments.

**Level of evidence:** IV

## Introduction

### Background

People presenting for speciality care of non-traumatic upper extremity musculoskeletal symptoms often report the symptom of ‘weakness’. We formed the impression that when a patient says, ‘I feel weak’, it often seems to indicate pain rather than abnormal muscle or nerve function. The experience of ‘weakness’ in the absence of nerve or muscle pathophysiology seems to be a type of disinhibition, at least partly unconscious, related to the fact that the human mind tends to interpret pain during an activity as indicating that the activity is making the problem worse (Dekker et al., 2021; Teunis et al., 2022). This common unhelpful misinterpretation of symptoms makes people less comfortable and less capable (Cremers et al., 2021) and that incapability may be experienced and expressed as ‘weakness’. In other words, an expression of weakness may be a verbal cue about unhelpful thoughts and feelings of distress, which are known to be associated with greater pain intensity and greater level of incapability (Crijns et al., 2022; Hadlandsmyth et al., 2017; Hageman et al., 2014; Howard et al., 2017; Kopp et al., 2021; Özkan et al., 2017; Ring et al., 2006; Rohrback et al., 2022).

### Rationale

There is a potential for a clinician to hear the word ‘weak’ and then to focus on the possibility of neuromuscular pathology. Although it is routine practice for hand specialists to examine for weakness, if the symptom of weakness is, at least in part, an expression of distress and unhelpful thinking about sensations, then focusing on the possibility of neuromuscular pathology could contribute to underappreciation and underdiagnoses of these aspects of the disorder. This may lead to unhelpful tests and treatments with potential for iatrogenic, psychological and financial harm. We have studied ‘weakness’ as a symptom, meaning that a patient reports symptoms of weakness rather than the specialist finding actual weakness during an examination.

In a cross-sectional study of people seeking upper extremity speciality care, we asked:

- What factors, including unhelpful thoughts and feelings of distress about symptoms, are associated with feelings of weakness among people seeking care for non-traumatic upper extremity conditions?
- What factors are associated with level of capability, including intensity of feelings of weakness, among people seeking musculoskeletal speciality care for non-traumatic upper extremity conditions?
- What factors are associated with pain intensity, including intensity of feelings of weakness, among patients seeking care for non-traumatic upper extremity conditions?

## Methods

### Study design and setting

Between March and April 2022, we invited all English-speaking adult (aged 18 years and older) patients presenting with a non-traumatic musculoskeletal problem to one of several musculoskeletal speciality clinics in an urban area in the United States to participate in this Institutional Review Board-approved cross-sectional study. A research assistant invited patients to complete a set of questionnaires. Patients were excluded if they were illiterate or had cognitive deficiencies. Completion of the questionnaires was considered to be written informed consent.

All patients were asked to complete measures of intensity of symptoms of weakness, level of capability, pain intensity, symptoms of depression, distress and misconceptions regarding symptoms and demographics on a secure Health Insurance Portability and Accountability Act compliant Research Electronic Data Capture survey tool.

### Participants

We screened all new and return patients for eligibility. A total of 139 eligible patients started the questionnaire and 135 (97%) patients completed it. Surveys without measurements of the primary outcome (reports of ‘weakness’) were removed (*n* = 4).

Of the participants, 76 (565%) were women, 99 (70%) were self-described as of white race/ethnicity, 74 (55%) were married and 84 (62%) were university or postgraduate educated. The mean age was 57 years (range 38–66) and all participants had education to high school level or above.

### Outcome measures

Weakness intensity was quantified using an 11-point ordinal scale from 0 (no weakness) to 10 (most severe weakness imaginable).

Pain intensity was measured using the Numeric Rating Scale (NRS), an 11-point ordinal scale from 0 (no pain) to 10 (worst pain imaginable) (Ferreira-Valente et al., 2011).

Level of capability was measured using the Patient-Reported Outcome Measurement Information System (PROMIS) Physical Function (PF) Computer Adaptive Test (CAT). PROMIS questionnaires are scaled to the general population of the United States, with a mean population score of 50. Every 10 points above or below 50 represents 1 standard deviation (SD). A higher score indicates a higher level of capability (Schalet et al., 2016a).

Symptoms of depression were measured using the PROMIS Depression CAT. A higher score indicates more symptoms of depression (Schalet et al., 2016b).

Unhelpful thoughts and distress about symptoms were measured using questionnaires consisting of three questions derived from factor analysis (Teunis et al., 2022). Each question was rated on a 5-point Likert scale from 1 (strongly disagree) to 5 (strongly agree).

### Statistical analysis

We performed descriptive statistics of all variables. Mean and SD were used for continuous variables with a normal distribution and median with interquartile range (IQR) for continuous variables with a non-parametric distribution. Categorical data were described as percentages with numbers.

In the bivariate analysis, we used the Mann–Whitney U test and Kruskal–Wallis H test for non-parametric continuous data and the Student’s *t*-test and one-way analysis of variance (ANOVA) for parametric continuous data to identify factors associated with reports of upper extremity weakness. Spearman’s correlation coefficient was calculated to assess the association between continuous explanatory variables and reports of upper extremity weakness, PROMIS PF CAT and NRS Pain intensity score. All variables with a *p-*value <0.10 in the bivariate analysis were moved to multivariable analysis.

A multiple linear regression model was used to identify factors associated with reports of upper extremity weakness and the NRS Pain Intensity Score. To identify factors associated with the PROMIS PF CAT we used a negative binominal regression model. All variables with a *p-*value <0.05 were considered statistically significant. We used PROMIS Depression and distress regarding symptoms to represent mental health, owing to concerns about multicollinearity with misconceptions regarding symptoms, which was excluded from multivariable analysis.

Owing to the low numbers, we pooled subgroups with fewer than 10 observations and randomly assigned non-binary individuals to either male or female categories for the purposes of analysis.

In an a priori sample size calculation we determined that 130 patients would provide <80% statistical power based on a multivariable linear regression model with 13 explanatory variables, if mental health factors account for 10% or more of the variation in reports of upper extremity weakness and if the complete model accounts for 25% or more of the overall variability (α = 0.05).

## Results

### Intensity of feelings of weakness

Accounting for potential confounding among factors brought forward from the bivariate analysis, including race/ethnicity marital status, and with selective inclusion of mental health measure to limit the potential for multicollinearity, a multiple linear regression analysis identified the following factors associated with greater intensity of feelings of weakness: greater distress regarding symptoms (regression coefficient [RC] 0.30; 95% confidence interval [CI]: 0.14 to 0.46; *p* < 0.001) and older age (RC 0.039; 95% CI: 0.011 to 0.66; *p* = 0.006). Male sex was associated with lower intensity of symptoms of weakness (RC −1.2; 95% CI: −2.0 to −0.41; *p* = 0.003).

### Factors associated with level of capability

Accounting for potential confounders such as sex, race/ethnicity, marital status and level of education, a negative binominal regression analysis identified the following factors associated with lower levels of capability: greater intensity of feelings of weakness (RC −0.029; 95% CI: −0.043 to −0.014; *p < *0.001), greater distress regarding symptoms (RC −0.024; 95% CI: = −0.037 to −0.010; *p* < 0.001), older age (RC −0.0023; 95% CI: −0.0045 to −0.00015; *p* = 0.036), but not greater symptoms of depression (*p* = 0.22). Insurance type ‘other’ (including Medicaid, workers’ compensation, uninsured and other) was modestly associated with greater level of capability relative to Medicare (RC 0.14; 95% CI: 0.033 to 0.24; *p* = 0.010).

### Factors associated with pain intensity

A multiple linear regression analysis identified the following factors associated with a higher NRS Pain Intensity Score: greater distress regarding symptoms (RC 0.34; 95% CI: 0.19 to 0.49; *p* < 0.001) and greater intensity of feelings of weakness (RC 0.20; 95% CI: 0.045 to 0.36; *p* = 0.012).

## Discussion

The word choice of patients can reflect unhelpful thoughts or feelings of distress about symptoms. We noticed that patients presenting with non-traumatic unilateral upper extremity conditions tend to use the word ‘weakness’ when describing discomfort and incapability. Perceived ‘weakness’ may be related to the common unhelpful perception that one needs to avoid painful activities to prevent making the problem worse, and the associated worry or despair related to a sense that one may lose the ability to rely on the hand. We found that the intensity of feelings of weakness was associated with distress regarding symptoms and that both the level of capability and pain intensity are independently associated with a greater intensity of feelings of weakness and more distress regarding symptoms. These findings suggest that clinicians can use the expression of feelings of weakness as a clue for potential unhelpful thinking or distress about symptoms and use that awareness to build a comprehensive care strategy that addresses those aspects of the disorder.

Distress (feelings of worry or despair) about symptoms is an important modifiable component of illness with a potential for improving health, given the association of distress with symptom intensity and level of capability (Cremers et al., 2021; Miner et al., 2021; Rohrback et al., 2022; Rossano et al., 2022). Studies of clinicians’ facial expressions confirm that they are aware of patients’ unhelpful thoughts and feelings of distress (Versluijs et al., 2021). Clinicians probably become aware of mindsets through specific words and postures that people use when they are experiencing unhelpful thoughts and feelings of distress (Bot et al., 2012; Wilkens et al., 2018). ‘Weakness’ can be added to the list of useful words that alert the clinician to opportunities to develop a healthier mindset in the patient.

The current study has some limitations. First, we acknowledge that most of our study population consisted of white and educated (high school or above) people living in an urban area in the United States. Therefore, our findings might be less applicable to other populations, and the means and rates need to be considered as context specific. However, the associations with mental health factors detected in this study should be reproducible in any sample with sufficient variation in the measured variables. Second, asking people to rate weakness may be variably interpreted and the same is true for the word pain. In addition, weakness or ‘loss of strength’ are, in our experience, the words used by patients to describe their experience. Third, we did not study the correlation of the symptom of weakness with actual weakness, in part because subtle weakness is difficult to quantify and in part because of existing evidence shows that symptom intensity does not correlate with the severity of the pathophysiology.

Based on the findings of this study, clinicians caring for people with musculoskeletal symptoms can use the symptom of weakness as a clue that a person may be experiencing distress about symptoms. The specialist can examine for weakness indicative of nerve and muscle disorders, but patients will also benefit from specialist identification and acknowledgement of their feelings about their symptoms, which could aid the establishment of trust and help the patient to feel heard and understood. When trust is established, it may be possible to start a conversation about mental health support. Some patients might be interested in developing a healthier mindset along with efforts to address any identified physical abnormality.
